# Supplementary material for: Modeling glioblastoma heterogeneity as a dynamic network of cell states
Source: Mol Syst Biol. 2021 Sep 16;17(9):e10105. doi: 10.15252/msb.202010105 (PMC8444284; doi:10.15252/msb.202010105)
Supplement: Supplementary file 6 — Source Data for Figure 5 [file MSB-17-e10105-s004.zip › Figure5A_sourcedata/GSEA_3017/hallmarks_stateF.GseaPreranked.1621934628066/HALLMARK_XENOBIOTIC_METABOLISM.html]

Details for gene set HALLMARK\_XENOBIOTIC\_METABOLISM[GSEA]

|  || Dataset | state33017 |
| Phenotype | NoPhenotypeAvailable |
| Upregulated in class | na\_pos |
| GeneSet | HALLMARK\_XENOBIOTIC\_METABOLISM |
| Enrichment Score (ES) | 0.42969823 |
| Normalized Enrichment Score (NES) | 2.0758412 |
| Nominal p-value | 0.0 |
| FDR q-value | 0.0032264465 |
| FWER p-Value | 0.026 |
Table: GSEA Results Summary

  

Fig 1: Enrichment plot: HALLMARK\_XENOBIOTIC\_METABOLISM      
 Profile of the Running ES Score & Positions of GeneSet Members on the Rank Ordered List

  

| PROBE | GENE SYMBOL | GENE\_TITLE | RANK IN GENE LIST | RANK METRIC SCORE | RUNNING ES | CORE ENRICHMENT || 1 | APOE |  |  | 17 | 1.244 | 0.0609 | Yes |
| 2 | NQO1 |  |  | 53 | 0.918 | 0.0956 | Yes |
| 3 | CBR1 |  |  | 87 | 0.779 | 0.1236 | Yes |
| 4 | PROS1 |  |  | 123 | 0.708 | 0.1468 | Yes |
| 5 | ALDH3A1 |  |  | 132 | 0.694 | 0.1814 | Yes |
| 6 | HMOX1 |  |  | 142 | 0.684 | 0.2150 | Yes |
| 7 | NINJ1 |  |  | 182 | 0.610 | 0.2310 | Yes |
| 8 | ATP2A2 |  |  | 210 | 0.582 | 0.2509 | Yes |
| 9 | PDLIM5 |  |  | 223 | 0.572 | 0.2770 | Yes |
| 10 | BLVRB |  |  | 235 | 0.565 | 0.3032 | Yes |
| 11 | CA2 |  |  | 243 | 0.558 | 0.3308 | Yes |
| 12 | ID2 |  |  | 366 | 0.483 | 0.3023 | Yes |
| 13 | CYFIP2 |  |  | 374 | 0.480 | 0.3256 | Yes |
| 14 | TGFB2 |  |  | 390 | 0.474 | 0.3449 | Yes |
| 15 | COMT |  |  | 396 | 0.471 | 0.3686 | Yes |
| 16 | GCNT2 |  |  | 413 | 0.465 | 0.3870 | Yes |
| 17 | EPHX1 |  |  | 456 | 0.448 | 0.3927 | Yes |
| 18 | CROT |  |  | 460 | 0.447 | 0.4160 | Yes |
| 19 | LPIN2 |  |  | 488 | 0.438 | 0.4279 | Yes |
| 20 | ALDH2 |  |  | 574 | 0.407 | 0.4120 | Yes |
| 21 | PINK1 |  |  | 639 | 0.387 | 0.4045 | Yes |
| 22 | GABARAPL1 |  |  | 654 | 0.384 | 0.4193 | Yes |
| 23 | TNFRSF1A |  |  | 686 | 0.374 | 0.4259 | Yes |
| 24 | FAS |  |  | 740 | 0.361 | 0.4219 | Yes |
| 25 | TPST1 |  |  | 767 | 0.354 | 0.4297 | Yes |
| 26 | GSTM4 |  |  | 909 | 0.324 | 0.3839 | No |
| 27 | ACOX1 |  |  | 912 | 0.324 | 0.4009 | No |
| 28 | DHRS7 |  |  | 1079 | 0.296 | 0.3423 | No |
| 29 | SMOX |  |  | 1305 | 0.268 | 0.2556 | No |
| 30 | ACO2 |  |  | 1443 | 0.253 | 0.2077 | No |
| 31 | GSTO1 |  |  | 1525 | -0.265 | 0.1858 | No |
| 32 | TMEM97 |  |  | 1629 | -0.293 | 0.1555 | No |
| 33 | HES6 |  |  | 1632 | -0.294 | 0.1708 | No |
| 34 | DCXR |  |  | 1689 | -0.315 | 0.1629 | No |
| 35 | UPP1 |  |  | 1726 | -0.328 | 0.1647 | No |
| 36 | PTGES3 |  |  | 1970 | -0.467 | 0.0808 | No |
| 37 | SERPINE1 |  |  | 2161 | -0.843 | 0.0415 | No |
Table: GSEA details [plain text format]

  

Fig 2: HALLMARK\_XENOBIOTIC\_METABOLISM: Random ES distribution      
 Gene set null distribution of ES for **HALLMARK\_XENOBIOTIC\_METABOLISM**
